# Self-Protective Function of Post-Conflict Bystander Affiliation in Mandrills

**DOI:** 10.1371/journal.pone.0038936

**Published:** 2012-06-08

**Authors:** Gabriele Schino, Claudia Marini

**Affiliations:** 1 Istituto di Scienze e Tecnologie della Cognizione, Consiglio Nazionale delle Ricerche, Rome, Italy; 2 Dipartimento di Biologia e Biotecnologie “Charles Darwin”, Università Sapienza, Rome, Italy; German Primate Centre, Germany

## Abstract

**Background:**

Affiliative interactions exchanged between victims of aggression and individuals not involved in the original aggression (bystanders) have been observed in various species. Three hypothetical functions have been proposed for these interactions: consolation, self-protection and substitute reconciliation, but data to test them are scanty.

**Methodology/Principal Findings:**

We conducted post-conflict and matched control observations on a captive group of mandrills (*Mandrillus sphinx*). We found that victims often redirected aggression to bystanders, that they received most affiliation from those bystanders that were frequently the target of redirection, and that bystander affiliation reduced the likelihood of redirection. Bystander affiliation did not reduce the victim's distress (as measured by its scratching rates) and was not received primarily from kin/friends. Finally, bystander affiliation did not reduce the likelihood of renewed aggression from the original aggressor.

**Conclusions/Significance:**

These results provide support for the self-protection hypothesis but not for the consolation and substitute reconciliation hypotheses.

## Introduction

For group living animals such as most primates, aggression is not a dyadic affair. Not only does aggression often involve more than two individuals [1], but in the ensuing period it can also affect the behavior of uninvolved group mates. For example, uninvolved bystanders can show affiliative behaviors directed to any of the original contestants [2], [3] or to other bystanders [4], [5]. Conversely, the victim of the original aggression can redirect aggression or it can receive renewed aggression from bystanders [3], [6]. The complex chain of events that is kicked off by an initial aggressive episode is an integral part of the more general phenomenon of conflict management and has been the subject of an extensive research effort (for reviews see [7], [8]).

While a large part of the investigations of post-conflict behavior focused on reconciliation, i.e. an affiliative contact between former opponents, several studies have also examined the affiliative behaviors that, immediately after aggression, can be exchanged between the victim of the original aggression and bystanding group mates. Following the original interpretation of de Waal and van Roosmalen [9], affiliative contacts directed from bystanders to victims were generally interpreted as “consolation”, that is, as having the function of helping the victim cope with the negative consequences of aggression by reducing the associated distress (although direct evidence of this hypothetical function has been obtained only recently; [10]). Bystander initiated affiliation towards the victim was observed in apes but not in monkeys. Such difference was considered coherent with the cognitive requirements of consolation, which seems to require some form of empathic understanding of the distress experienced by the victim [11].

More recently, however, alternative functional interpretations have been proposed for bystander initiated post-conflict affiliation. Wittig et al. [12] showed that in chacma baboons (*Papio hamadryas ursinus*) affiliative interactions that the victim receives from the kin of the original aggressor can function as substitute of direct reconciliation. Call et al. ([13], in *Macaca arctoides*) and Koski & Sterck ([14], [15], in *Pan troglodytes*) suggested that bystander affiliation with victims of aggression has a preemptive function in reducing the probability of receiving redirected aggression. As noted by Fraser et al. [16], bystander affiliation directed to victims of aggression is likely to be a heterogeneous phenomenon serving different functions in different species and contexts.

Our understanding of the functional significance of bystander affiliation has been hindered by the paucity of relevant data. In fact, several of the studies that have reported on bystander affiliation did not even try to address functional interpretations or, when they did, they focused on only one of the possible functions (review in [16]). The aim of this study was to attempt a simultaneous evaluation of three hypothetical functions of post-conflict bystander affiliation in mandrills (*Mandrillus sphinx*). After showing that bystanders do engage in increased affiliation with victims of aggression we tested the following predictions.

### Consolation hypothesis

If bystander affiliation functions to console victims of aggression, than we expect that: 1) bystander affiliation should be received primarily from kin and/or friends (i.e., individuals exchanging frequent grooming); 2) bystander affiliation should reduce the frequency of scratching (a behavioral indicator of stress and anxiety [17], [18]); 3) bystander affiliation should be more likely after intense aggression.

### Self-protection hypothesis

If bystander affiliation functions to protect the bystander from the risk of receiving redirected aggression, than we expect that: 1) redirected aggression should be common; 2) bystander affiliation should be received primarily from individuals that are frequently the target of redirection; 3) bystander affiliation should be received primarily from individuals ranking lower than the victim (that are presumably more at risk); 4) bystander affiliation should reduce the likelihood of redirection.

### Substitute reconciliation hypothesis

If bystander affiliation functions as a substitute of direct reconciliation between victim and aggressor, then we expect that: 1) bystander affiliation should be both received and directed by/to kin of the aggressor; 2) bystanders offering affiliation should be more closely related to aggressors than to victims, because kin of the aggressor will be most able to contribute to repairing the aggressor-victim relationship [19]; 3) post-conflict affiliation received from a kin of the aggressor should reduce the likelihood of renewed aggression by the former aggressor.

## Methods

### Ethical Statement

This study was conducted in accordance with Italian legislation, which does not require purely observational studies to be approved by an ethic committee.

### Subjects and Housing

The mandrills that served as subjects of this study lived in the Rome zoo (Bioparco) in a 240 m^2^ outdoor enclosure connected with indoor quarters. Our study group included three sexually mature males (one adult and two subadults), seven mature females, three juveniles (one female and two males) and one infant. At the beginning of the study, two more mature females were present in the group, but they were removed for management purposes about one month after the beginning of data collection.

We obtained degrees of maternal kinship (ranging from 0.125 to 0.5) from demographic records. The alpha male and one of the adult females had no maternal relatives. All other individuals belonged to one of three matrilines.

### Data Collection

C.M. collected data from May to December 2009, between 9.00 and 17.30 (excluding feeding time), following the PC-MC method of de Waal & Yoshihara [20]. Aggressive interactions included both contact aggression (biting or grabbing) and non-contact aggression (staring, open-mouth, head-bob, ground-slap, chasing; see Table S1 for definitions). For each aggression we recorded its intensity (with or without physical contact) and the aggressor and victim identities. Post-conflict focal animal observations (PCs) were conducted on the victim immediately after the end of the aggressive interaction. If aggression resumed between the same subjects within 30 s from the original aggression the observation was aborted and started again when aggression terminated. If the subject went out of view (for example, because it entered the indoor quarters), the observation was interrupted. Data collected up to the interruption were included into the analyses. A matched control observation (MC) was made on the same focal subject using an identical procedure on the next possible observation day, at approximately the same time of day, under similar weather conditions. Both PC and MC observations lasted 10 minutes. During PC and MC observations, we recorded all affiliative and aggressive interactions in which the victim of the initial aggression was involved, as well as the identity of its partners (see Table S1 for a list of the behavior patterns and their definitions).

A total of 576 PC-MC pairs were recorded. Twelve different subjects were sampled (median = 43.5 PC-MC pairs per subject, range 5–102). The alpha male was never sampled as a focal subject because it never received an aggression. Data were also not collected on the single infant in the group, and the few observations that had been conducted on the two females that were removed from the group were excluded from analyses.

We also conducted 92.5 hours of focal group observations (i.e., observations in which the behavior of all group members was recorded) on grooming and aggression in order to obtain dyadic data of time spent grooming and frequency of aggression.

In order to determine the dominance hierarchy of the group we collapsed data collected during focal group and matched control observations, supplemented by *ad libitum* data collected opportunistically.

### Data Analysis

Animals were arranged in a linear dominance hierarchy by minimizing the number of occurrences below the diagonal in a matrix of dyadic unidirectional agonistic interactions. Landau's linearity index was h′ = 0.690, P<0.001.

We adopted survival analysis (the logrank test) to test whether affiliation or aggression between victims and group members not involved in the original aggression (hereafter, bystanders) occurred sooner in PC than in MC observations [21]. We entered the identity of the victim as a “stratification” variable in order to avoid pseudoreplication [22]. In order to provide information comparable to that of previous studies, we also calculated triadic contact tendencies (TCT) and redirection tendencies (RT) adapting the measure proposed by Veenema et al. [23]. We calculated individual TCTs and RTs and present their means and standard errors.

In order to identify the time window during which affiliative interactions between victims and bystanders or redirected aggression from victims to bystanders were more frequent in PC than in MC observations, we carried out negative binomial regressions for count data in which the number of affiliative interactions or of aggressive interactions were the dependent variables and the duration of the observation was the “exposure” variable. We compared rates of affiliation and of aggression in each PC minute with those during whole MC observations. We also entered the identity of the victim as a fixed effect variable in order to avoid pseudoreplication. Entering the subject identity as a fixed effect independent variable is mathematically equivalent to carrying out within-subject centering [24] and thus allows controlling for between subject variations. The same analysis was used to compare rates of scratching in PC and MC observations.

For each individual, we calculated its probability of receiving post-conflict affiliation from or of redirecting aggression to any other group member. Such probabilities were calculated obtaining the number of affiliative interactions received or redirected aggression given that had occurred within the relevant time window (identified as explained above) and then dividing this figure for the number of times the subject had been sampled as a victim (i.e., the occasions the subject had of receiving post-conflict affiliation or of redirecting aggression). We also calculated, again for each dyad, the time spent grooming, as an index of the general affiliation characterizing it. We then entered dyadic scores into a within-subject linear regression with robust standard errors [25] in which the post-conflict increase in the probability of receiving affiliation (calculated as the difference between the probabilities obtained from PC and MC observations) was the dependent variable, and kinship, time spent grooming, the probability of redirecting aggression, and whether the victim was higher ranking than the bystander were the independent variables.

We also used a within-subject conditional logistic regression to test whether the intensity of the aggression received influenced the probability of receiving post-conflict affiliation from a bystander.

Finally, we evaluated the effects of post-conflict bystander affiliation on the rate of redirected aggression by means of a Wilcoxon matched-pairs signed-ranks test. For each bystander, we calculated the rate of redirected aggression it received after it had directed an affiliative interaction to the victim and in the absence of such interaction. Data points entered into this analysis were thus averages for each subject. For PC observations in which bystander-victim affiliation had occurred, we included into analysis the time window between affiliation and the end of the 5th minute post-conflict (since an increase in redirected aggression was detectable for five minutes after the initial aggression; see the Results). For PC observations in which bystander-victim affiliation had not occurred, we included into analysis the time window between the average timing of affiliation (50 s) and the end of the 5th minute post-conflict. Note that this analysis compared redirected aggression received by bystanders in the presence versus absence of post-conflict bystander affiliation in comparable time windows after the initial conflict. Similar analyses were used to evaluate the effect of bystander affiliation on the victim scratching, and on the rate of renewed aggression received by the victim from the original aggressor.

Data were tested for normality using the Shapiro-Francia normality test and, when necessary, were transformed using either the arcsin (for proportion data) or the logarithmic (for grooming, aggression and scratching data) transformation. Data transformation reduced, but did not completely eliminate heteroscedasticity. We therefore obtained p values on the basis of robust standard errors [26]. All analyses were carried out using Stata 11.2 [27] and all reported probabilities are two tailed.

## Results

### Frequency of Grooming and Aggression

Mandrills spent on average 170±62 s/h grooming their group companions (mean and standard error). Average rate of aggression was 0.66±0.19 ep./h.

### Occurrence of Affiliation with Bystanders

**Figure 1:**
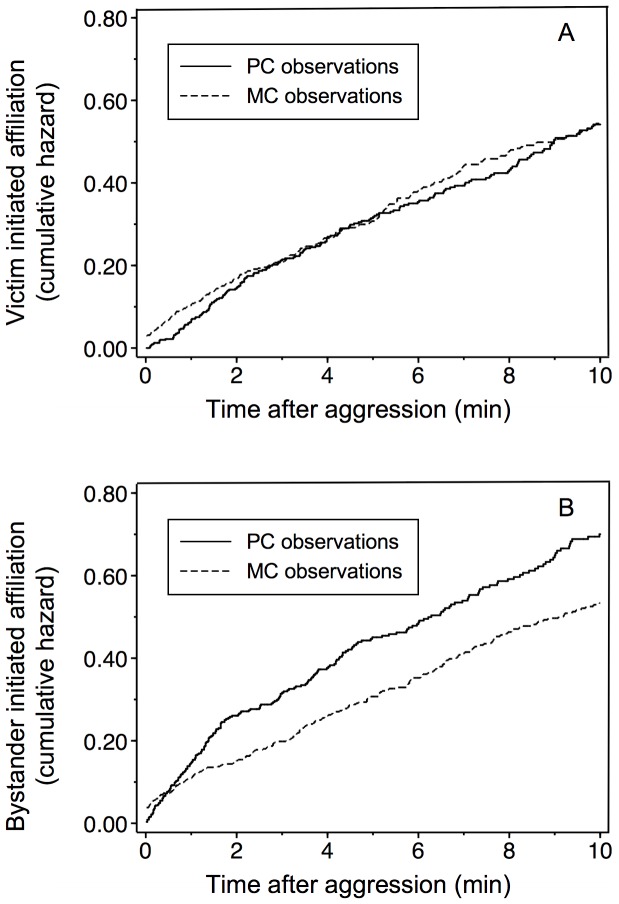
Time course of affiliation between victims and bystanders in post-conflict (PC) and matched control (MC) observations. Nelson-Aalen cumulative hazard (i.e., cumulative rate) of the first affiliative interaction between victims and bystanders as derived from a survival analysis. (a): affiliation initiated by the victim; (b): affiliation initiated by the bystander.

A survival analysis showed that victim-initiated affiliation did not occur earlier in post-conflict than in matched control observations (χ^2^ = 0.03, df = 1, P = 0.873; Fig. 1a). In contrast, affiliation initiated by bystanders towards victims occurred significantly earlier in PC than in MC observations (χ^2^ = 8.17, df = 1, P = 0.004; Fig. 1b). Negative binomial regressions showed that rates of bystander-initiated affiliation were higher in PC than in MC observations for the first two minutes post-conflict (Table 1). When the analysis was repeated lumping together data from the first two minutes of observation, the difference between post-conflict and control observations was significant (coefficient = 0.696, z = 5.93, P<0.001). Mean (±SE) triadic contact tendency was 0.071±0.046.

**Table 1:** Comparison of bystander-initiated affiliation with the victim of aggression in the 10 minutes of the post-conflict and matched control observations.

PC minute	Coefficient	z value	N	P value
1	0.714	5.27	1152	<0.001
2	0.640	4.46	1104	<0.001
3	0.083	0.48	1072	0.633
4	0.070	0.39	1045	0.694
5	0.410	2.51	1023	0.012
6	−0.069	−0.35	999	0.727
7	0.172	0.93	975	0.353
8	−0.057	−0.28	962	0.778
9	0.210	1.12	945	0.263
10	0.024	0.12	931	0.905

Results of negative binomial regressions comparing the rates of bystander-initiated affiliation with the victim of aggression in post-conflict (PC) and matched control (MC) observations. Each PC minute is compared with the whole MC observation.

### Post-conflict Anxiety

**Figure 2:**
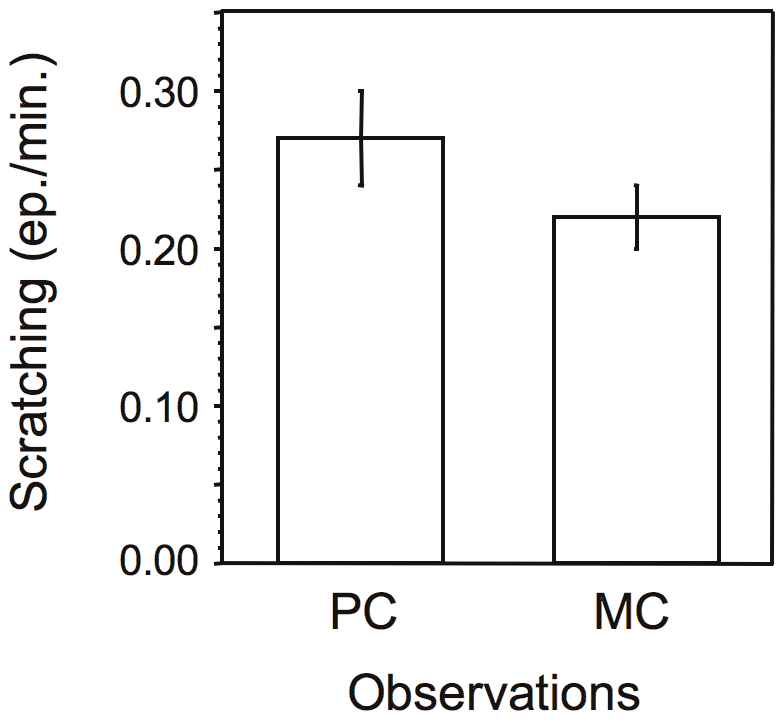
Rate of scratching in post-conflict (PC) and matched control (MC) observations. Means and standard errors calculated over the first eight minutes of observation (see the text for details).

Although somewhat inconsistently, mandrills showed an increase in the rate of scratching (a behavioral indicator of stress or anxiety) following the receipt of aggression that extended for the first eight minutes post-conflict (Table 2). When the analysis was repeated lumping together data from the first eight minutes of observation, the difference between post-conflict and control observations was significant (coefficient = 0.158, z = 2.69, P = 0.007; Fig. 2).

**Table 2:** Comparison of the rates of scratching by the victim of aggression in the 10 minutes of the post-conflict and matched control observations.

PC minute	Coefficient	z value	N	P value
1	0.171	1.82	1152	0.069
2	0.196	2.07	1104	0.039
3	−0.006	−0.05	1072	0.957
4	0.119	1.16	1045	0.246
5	0.093	0.88	1023	0.377
6	0.222	2.14	999	0.032
7	0.186	1.74	975	0.083
8	0.213	1.98	962	0.047
9	−0.195	−1.51	945	0.132
10	−0.023	−0.19	931	0.849

Results of negative binomial regressions comparing the rates of scratching by the victim of aggression in post-conflict (PC) and matched control (MC) observations. Each PC minute is compared with the whole MC observation.

### Test of the Consolation Hypothesis

Contrary to the predictions of the consolation hypothesis, post-conflict affiliation was not received primarily from kin, was not related to the dyadic grooming score (Table 3) and was not more likely to occur after more intense aggression (within-subject logistic regression: coefficient = −0.014, z = −0.06, P = 0.948). Also, receiving post-conflict affiliation did not reduce the rate of scratching (Wilcoxon matched-pairs signed-ranks test: T = 15, N = 11, N.S.).

**Table 3:** Factors affecting the probability of post-conflict bystander affiliation.

Variable	Coefficient	t value	P value
Kinship	−0.0045	−0.10	0.919
Dyadic grooming score	−0.163	−1.88	0.089
Prob. of redirection	0.3008	2.50	0.031
Relative rank	−0.0241	−1.34	0.210
Intercept	0.0449	2.22	0.050

df = 10 in all tests. The overall model is significant (F = 3.71, df = 4,10, P = 0.042; N = 132 dyads).

### Test of the Self-protection Hypothesis

**Figure 3:**
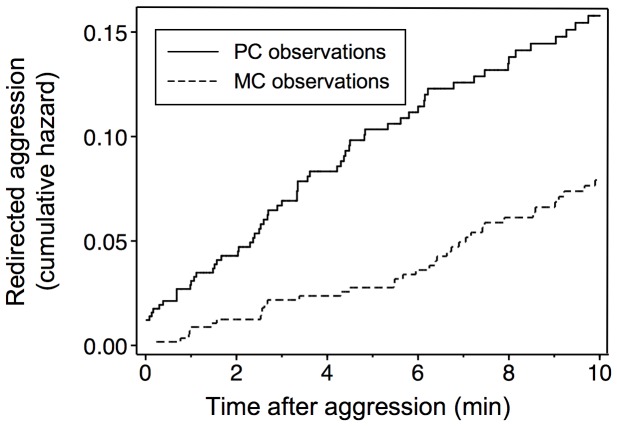
Time course of redirected aggression in post-conflict (PC) and matched control (MC) observations. Nelson-Aalen cumulative hazard (i.e., cumulative rate) of the first aggressive interaction directed by the victims of the original aggression to a bystander as derived from a survival analysis.

Confirming the predictions of the self-protection hypothesis, victims of aggression often redirected aggression to bystanders. A survival analysis showed that victim-initiated aggression occurred earlier in PC than in MC observations (χ^2^ = 16.01, df = 1, P = 0.0001; Fig. 3). Negative binomial regressions showed that rates of redirected aggression were higher in PC than in MC observations for the first five minutes post-conflict (Table 4). When the analysis was repeated lumping together data from the first five minutes of observation, the difference between post-conflict and control observations was significant (coefficient = 1.106, z = 4.84, P<0.001). Mean (±SE) redirection tendency was 0.043±0.016.

**Table 4:** Comparison of the rates of redirected aggression by the victim of aggression to a bystander in the 10 minutes of the post-conflict and matched control observations.

PC minute	Coefficient	z value	N	P value
1	1.534	5.69	1152	<0.001
2	0.331	0.75	1104	0.450
3	1.018	2.99	1072	0.003
4	0.752	1.94	1045	0.052
5	0.915	2.48	1023	0.013
6	0.709	1.73	999	0.084
7	0.204	0.39	975	0.697
8	0.642	1.46	962	0.143
9	0.305	0.58	945	0.561
10	0.743	1.69	931	0.090

Results of negative binomial regressions comparing the rates of redirected aggression by the victim of aggression to a bystander in post-conflict (PC) and matched control (MC) observations. Each PC minute is compared with the whole MC observation.

Also, post-conflict affiliation was most often received from those individuals that were frequently the target of redirected aggression, although not by individuals that were lower-ranking than the victim (Table 3).

Finally, directing affiliation to the victim of the original aggression reduced the probability of receiving redirected aggression (T = 0, N = 11, P<0.01). In fact, not a single episode of redirected aggression was observed being received by a bystander after its affiliation with the victim of the original aggression.

### Test of the Substitute Reconciliation Hypothesis

Contrary to the predictions of the substitute reconciliation hypothesis, affiliation was not both directed and received by victims of the original aggression to/from the kin of the original aggressor. Survival analysis showed that affiliation was received by victims from kin of the aggressor earlier in PC than in MC observations, just like in the analysis of the complete sample (χ^2^ = 3.77, df = 1, P = 0.052). However, affiliation was not directed by victims to the kin of the aggressor earlier in PC than in MC observations (χ^2^ = 0.02, df = 1, P = 0.897).

Also, degrees of kinship between bystanders offering post-conflict affiliation and aggressors were not higher than those between bystanders and victims (within-subject regression: coefficient = −0.038, t = −0.44, df = 10, P = 0.667).

Finally, receiving affiliation from a kin of the aggressor did not reduce the rate of renewed aggression by the original aggressor (T = 9, N = 9, N.S.).

## Discussion

Following aggression, mandrill victims received increased affiliation from bystanders (a phenomenon rarely reported in monkeys). Of the three functional hypotheses we tested, the self-protection hypothesis provided the best fit to the data. Victims redirected aggression frequently to bystanders, affiliation was received primarily from those bystanders that were more often the target of redirection, and affiliation was associated to a reduction in the probability of redirection. Although this latter result does not necessarily imply a causal relation between bystander affiliation and a reduction in redirection, this interpretation is supported by the observation that the average latency of bystander affiliation (50 s) was shorter than that of redirection (121 s). In contrast, our data did not support either the consolation or the substitute reconciliation hypotheses.

Among primates, affiliation directed from bystanders to the victim of aggression has been observed almost exclusively in apes, while in monkeys post-conflict affiliation with bystanders is generally initiated by the victim ([3]; for a single exception see [13]). Given this taxonomic bias, bystander affiliation has been traditionally interpreted as consolation and the ape/monkey difference as due to differences in the cognitive capacity for empathy, considered a necessary prerequisite for consolation. This interpretation, however, has been called into question by the observation of bystander affiliation to the victim of aggression in non-primate species whose capacity for empathy is unclear (corvids: [28], [29]; canids: [30], [31]; see [32] for a discussion of the cognitive requirements of the different degrees of empathy).

More recently, alternative functional interpretations of bystander affiliation have been proposed, and it is becoming increasingly clear that bystander affiliation is a heterogeneous phenomenon. In this regard, a paradigmatic example is provided by studies of bystander affiliation in chimpanzees, a species whose capacity for some form of empathy is undisputed [33]. Detailed functional analyses of chimpanzee post-conflict behavior have shown that in this species bystander affiliation may function as consolation [10], [34], [35], as self-protection [15] and as substitute reconciliation [19].

The next step, of course, is to attempt an explanation of the observed inter- and intra-specific variability in bystander affiliation. First, we know that the different species differ in the constraints that are imposed on the possible uses of bystander affiliation. Species whose cognitive capacities do not allow empathic understanding of the need for distress alleviation experienced by other individuals cannot possibly use bystander affiliation as consolation even when this would be functionally valuable. For example, Japanese macaque (*Macaca fuscata*) mothers failed to affiliate with their offspring after the latter had received aggression even if kin selection made them the most likely candidate to do so [36]. Similarly, the use of bystander affiliation as substitute reconciliation requires some understanding of third-party social relationships. This is likely within the cognitive capacities of most primate species [37], but it is still unknown with regard to other species (e.g., canids) that do show bystander affiliation.

A factor that can potentially help to explain both intraspecific and interspecific variation is the frequency and intensity of redirected aggression. Generally speaking, bystander affiliation would be more valuable as a self-protection strategy when redirected aggression is more common or more intense. Unfortunately, we do not have sufficient comparative data to ascertain if, for example, chimpanzees living in groups that experience higher redirected aggression use bystander affiliation as a self-protection strategy more often than conspecifics living in groups where redirected aggression is less common. A full understanding of the variations in the prevalence and function of bystander affiliation will require detailed comparative data on the context and short-term consequences of aggression. The results of our study add to the available information by showing that bystander affiliation is common in a monkey species and by providing clear evidence for one of its hypothetical functions. Our results also emphasize the need to interpret bystander affiliation in the context of the various short-term consequences of aggression, that include both affiliative (reconciliation: [38]) and aggressive events (redirection: this study).

## Supporting Information

Table S1 Behaviors recorded during post-conflict and matched control observations.
(PDF)
