# Supplementary material for: Self-Protective Function of Post-Conflict Bystander Affiliation in Mandrills
Source: PLoS One. 2012 Jun 8;7(6):e38936. doi: 10.1371/journal.pone.0038936 (PMC3371020; doi:10.1371/journal.pone.0038936)
Supplement: Table S1 — Behaviors recorded during post-conflict and matched control observations. (PDF) [file pone.0038936.s001.pdf]

Supplementary Information for:

Self-Protective Function of Post-Conflict Bystander Affiliation in Mandrills

Gabriele Schino and Claudia Marini

Table S1. Behaviors recorded during post-conflict and matched control observations

**Behavior**	**Definition**
Aggressive behaviors	
Threat	staring, open-mouth, head-bob (the head and possibly the upper body is thrusted forward and down), ground-slap
Chase	meaning obvious
Physical assault	aggression involving physical contact (biting or grabbing)
Affiliative behaviors	
Allogrooming	careful picking and/or slow brushing aside the fur of a partner with one or both hands and/or the mouth
Genital inspect	touching and/or sniffing the anogenital region of a partner
Hand touch	briefly touching a partner
Mounting	dorsoventral mounting, with or without double foot clasp
Muzzle contact	contacting the muzzle of a partner with one own's muzzle
Social play	rough and tumble play
Sitting in contact	sitting with the body in contact with a partner
Bared teeth	the lips are curled back in a horizontal figure eight shape, revealing the canines and premolars
Crest raise	the crest is erected while the head is shaken repeatedly from side to side
